# No evidence for an association between the -871 T/C promoter polymorphism in the B-cell-activating factor gene and primary Sjögren's syndrome

**DOI:** 10.1186/ar1884

**Published:** 2006-01-09

**Authors:** Jacques-Eric Gottenberg, Jérémie Sellam, Marc Ittah, Frédéric Lavie, Alexis Proust, Habib Zouali, Christelle Sordet, Jean Sibilia, Robert P Kimberly, Xavier Mariette, Corinne Miceli-Richard

**Affiliations:** 1 Rhumatologie, INSERM E 109, Hôpital Bicêtre, Assistance Publique-Hôpitaux de Paris, Université Paris-Sud 11, Le Kremlin Bicêtre, France; 2 Hématologie, INSERM E 109, Hôpital de Bicêtre, Assistance Publique-Hôpitaux de Paris (AP-HP), Le Kremlin Bicêtre, France; 3 Fondation Jean Dausset, Centre d'Etude du Polymorphisme Humain(CEPH), Paris, France; 4 Rhumatologie, Centre Hospitalier Universitaire de Strasbourg, Strasbourg, France; 5 Division of Clinical Immunology and Rheumatology, University of Alabama, Birmingham, AL, USA

## Abstract

Polyclonal B cell activation might be related to pathogenic over-expression of B-cell-activating factor (BAFF) in primary Sjögren's syndrome (pSS) and other autoimmune diseases. We therefore investigated whether BAFF over-expression in pSS could be a primary, genetically determined event that leads to the disease. The complete BAFF gene was sequenced in Caucasian pSS patients and control individuals. The only single nucleotide polymorphism frequently observed, namely -871 T/C in the promoter region, was then genotyped in 162 French patients with pSS and 90 French control individuals. No significant differences in allele (T allele frequency: 49.7% in patients with pSS versus 50% in controls; *P *= 0.94) and genotype frequencies of BAFF polymorphism were detected between pSS patients and control individuals. BAFF gene polymorphism was not associated with a specific pattern of antibody secretion either. T allele carriers had significantly increased BAFF protein serum levels (mean values of 8.6 and 5.7 ng/ml in patients with TT and TC genotypes, respectively, versus 3.3 ng/ml in patients with CC genotype; *P *= 0.01), although no correlation was observed between BAFF polymorphism and mRNA level. In conclusion, BAFF gene polymorphism is neither involved in genetic predisposition to pSS nor associated with a specific pattern of antibody production.

## Introduction

Polyclonal B-cell activation might be related to pathogenic over-expression of B-cell-activating factor (BAFF; also known as TNFSF13B, BLyS, THANK and TALL-1) in primary Sjögren's syndrome (pSS) and other autoimmune diseases [1]. In pSS, an increase in serum BAFF level was reported in all published surveys of patients with pSS [2-5]. In addition, we and others [4-6] have demonstrated increased BAFF expression in salivary glands, the main target of this autoimmune disease. We hypothesized that BAFF over-expression in pSS could be a primary, genetically determined event that leads to the disease. We therefore investigated the genetic association between BAFF polymorphisms and pSS.

## Materials and methods

Because BAFF polymorphisms have never been studied in Caucasian patients, the complete BAFF gene was sequenced in 13 patients with pSS and 13 healthy control individuals. Two single nucleotide polymorphisms (SNPs) were detected in the promoter region of the BAFF gene: -661 A/G, a rare variation observed in only one healthy individual; and -871 T/C, which was observed among patients with pSS with a C allele frequency of 46%. Five other SNPs, previously reported in Japanese patients with rheumatoid arthritis (RA) and systemic lupus erythematosus (SLE) [7], were not detected in the present study of Caucasian patients. After isolation of genomic DNA from peripheral blood mononuclear cells (PBMCs), the -871 T/C SNP was genotyped using PCR restriction fragment length polymorphism method in 162 unrelated French patients with pSS (110 patients with anti-SSA and/or anti-SSB autoantibodies and 52 patients without autoantibodies). Patients were defined in accordance with European–American consensus group criteria and were recruited from the Departments of Rheumatology of Bicêtre and Strasbourg Hospitals. Ninety healthy French blood donors were genotyped as controls. All patients and control individuals were Caucasians. The characteristics of the patients are summarized in Table 1. The patients were receiving no immunosuppressive medications other than corticosteroids (daily dose of ≤10 mg of prednisone; *n *= 14) or hydroxychloroquine (*n *= 13). Of the 162 patients, 95 were included in a previous study in which BAFF level was reported [8]. The methods of assessment of serum BAFF using enzyme-linked immunosorbent assay were previously reported [2].

Levels of BAFF mRNA were determined by real-time quantitative PCR using a LightCycler (Roche Diagnostics, Manheim, Germany). PBMCs were isolated from 20 ml venous blood samples from 40 patients with pSS and stored at -70°C in RNAlater (Qiagen, Valencia, CA). Total RNA was extracted from PBMCs using RNeasy Mini Kit (Qiagen). The cDNA synthesis was performed using Enhanced Avian HS RT-PCR (Sigma-Aldrich, Saint Quentin Fallavier, France). BAFF and β-actin mRNA levels were assessed by real-time quantitative PCR using the following primers: 5'-TGAAACACCAACTATACAAAAAG-3' and 5'-TCAATTCATCCCCAAAGACAT-3' for BAFF; and 5'-GCTGTGCTACGTCGCCCT-3' and 5'-AAGGTAGTTTCGTGGATGCC-3' for β-actin. Primers were designed to be specific for full-length BAFF, excluding any amplification of ΔBAFF. Each sample was run with initial incubation at 96°C for 10 minutes, and thermal conditions followed 40 cycles at 95°C for 10 s, 60°C for 15 s and 72°C for 20 s. For each run, serially diluted cDNA of K562 cells was used as a quantitative standard. To correct for variations in mRNA recovery and the reverse transcription yield, the amounts of BAFF cDNA were normalized to β-actin.

Genotypic and allelic frequencies were compared by χ^2 ^testing. The association between BAFF polymorphism and BAFF protein or mRNA level was analyzed using the Mann–Whitney U test. The association between BAFF polymorphism and serum gammaglobulin, IgG, and rheumatoid factor levels was analyzed using analysis of variance. Statistical analysis was performed using Analyse-it for Microsoft Excel (Leeds, England, UK).

## Results

The allelic and genotypic distribution of -871 T/C polymorphism were in Hardy–Weinberg equilibrium. No significant differences in allele and genotype frequencies of BAFF polymorphism were detected between patients with pSS and control individuals (T allele frequency: 49.7% in patients with pSS versus 50% in controls, *P *= 0.94; TT genotype: 26% in pSS versus 23.3%; TC genotype: 48.5% versus 53.4%, CC genotype: 25.5% versus 23.3%, *P *= 0.78). No significant difference was observed in terms of clinical presentation (36 and 38% of extraglandular involvement in T and C allele carriers, respectively; *P *= 0.8). BAFF polymorphism was not involved in genetic predisposition to a specific pattern of autoantibody secretion either (T allele frequency in patients without autoantibody: 45%; in patients positive for anti-SSA autoantibody only: 48%; in patients positive for anti-SSA + anti-SSB autoantibody: 52%; *P *= 0.76). No association was observed between BAFF gene polymorphism and mean serum gammaglobulin, IgG, or rheumatoid factor levels assessed by nephelometry (gammaglobulin, IgG, and rheumatoid factor, respectively: 11.9 ± 0.7 g/l, 13.8 ± 1.1 g/l and 154.2 ± 75.2 IU/l in patients with CC genotype [*P *= 0.3]; 14.1 ± 1 g/l, 15.7 ± 1.2 g/l and 157.8 ± 32.4 IU/l in patients with TC genotype [*P *= 0.61]; and 12.7 ± 1 g/l, 14.9 ± 1.3 g/l, 267.7 ± 113.5 IU/l in patients with TT genotype [*P *= 0.43]).

**Figure 1:**
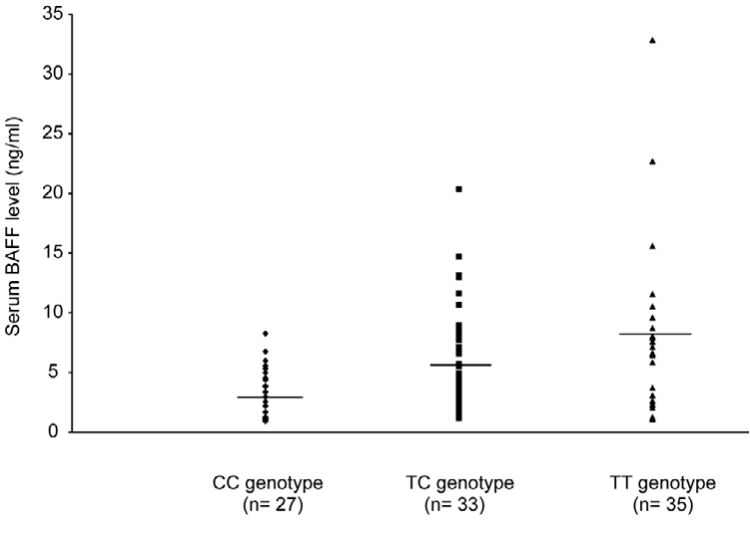
Association between BAFF -871 T/C polymorphism and serum BAFF level in 95 patients with pSS. Bars represent mean serum BAFF levels. BAFF = B-cell-activating factor; pSS, primary Sjögren's syndrome.

**Figure 2:**
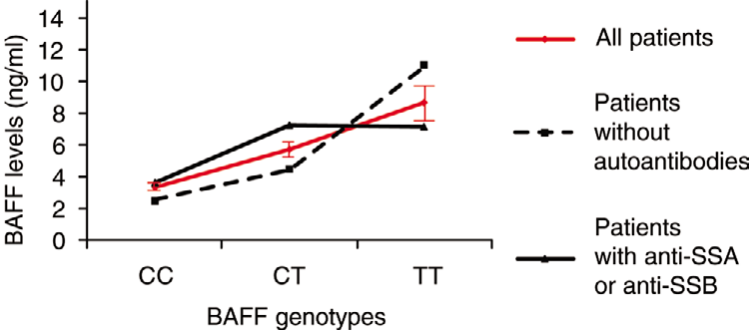
Association between BAFF -871 T/C polymorphism and serum BAFF level in 95 patients with pSS. The bar represents the standard error. BAFF = B-cell-activating factor; pSS, primary Sjögren's syndrome.

A significant association was observed between -871 T/C polymorphism and serum BAFF level: T allele carriers had a significantly higher BAFF level than did C allele carriers. Thus, mean BAFF levels were 8.6 ± 2 and 5.7 ± 0.6 ng/ml in patients with TT and TC genotypes versus 3.3 ± 0.4 ng/ml in patients with CC genotype (*P *= 0.01; Fig. 1). T allele was associated with increased BAFF levels in the 27 patients without autoantibodies (CC genotype [*n *= 7]: 2.5 ± 0.6 ng/ml; TC genotype [*n *= 13]: 4.5 ± 0.7 ng/ml; TT genotype [*n *= 7]: 11.6 ± 6.3 ng/ml; *P *= 0.08) and in the 68 patients with anti-SSA or anti-SSB antibodies (CC genotype [*n *= 16]: 3.6 ± 0.6 ng/ml; TC genotype [*n *= 31]: 6.1 ± 0.8 ng/ml; TT genotype [*n *= 21]: 7.1 ± 1.7 ng/ml; *P *= 0.05; Fig. 2). Serum BAFF level did not differ significantly according to the presence of concomitant treatment with low-dose corticosteroids (*n *= 14 [daily dose ≤10 mg in all patients]; median BAFF level: 4.3 ng/ml [95% confidence interval (CI) 2.7–6.5] versus 5.3 ng/ml [95% CI 2.9–15.6] in patients without corticosteroids; *P *= 0.76) or with hydroxychloroquine (*n *= 13; median BAFF level: 3.8 ng/ml [95% CI 1.7–8.7] versus 4.7 ng/ml [95% CI 3.1–7.1] in patients without hydroxychloroquine; *P *= 0.53).

**Figure 3:**
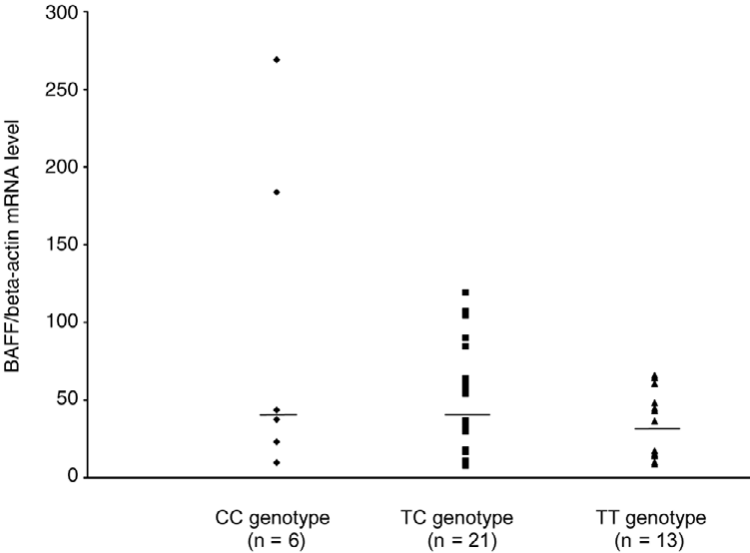
Association between BAFF -871 T/C polymorphism and BAFF mRNAin 40 patients with pSS. Bars represent median levels of BAFF mRNA. BAFF = B-cell-activating factor; pSS, primary Sjögren's syndrome.

The correlation between BAFF polymorphism and protein level led us to investigate whether a similar correlation could be found with BAFF mRNA level in PBMCs from patients with pSS. The median BAFF mRNA normalized level was 43.39 (25th to 75th percentile: 17–62.2). Two of the 40 patients had outlying BAFF/β-actin values of 269 and 183.8. These patients had no specific clinical features; the reassessment of BAFF mRNA levels in these patients confirmed these values. BAFF polymorphism was not associated with BAFF mRNA level; BAFF mRNA level was not significantly different between patients carrying -871 T allele and those not carrying -871 T allele (median normalized BAFF mRNA levels 36.8 [95% CI 29.6–54.1] and 40.5 [95% CI 9.6–269], respectively; *P *= 0.55; Fig. 3). When the two patients with outlying values for BAFF mRNA and CC genotype were not taken into account, a nonsignificant trend was observed toward an association between BAFF polymorphism and BAFF mRNA levels (median normalized BAFF mRNA level: 43.8 in patients carrying -871 T allele and 30.2 in patients with CC genotype; *P *= 0.24).

## Discussion

The present study is the first to investigate the genetic contribution of BAFF to pSS. Indeed, numerous data support a pathogenic role for BAFF in pSS, such as the phenotype of BAFF transgenic mice [9], which develop Sjögren's syndrome-like symptoms with age, and the increased serum and salivary expression of BAFF in patients with pSS [2-6]. Moreover, because one-third of first-degree relatives of patients with primary pSS suffer from other autoimmune diseases [10] and given that BAFF over-expression was also demonstrated in RA [11] and SLE [12], BAFF could be a good candidate gene in the predisposition to multiple autoimmune diseases, as was recently observed for the genes encoding PTPN22, RUNX1, PDCD1 and CTLA4 [13]. The findings presented here demonstrate that BAFF gene polymorphism is associated neither with predisposition to pSS nor with a specific pattern of antibody secretion, including anti-SSA/SSB autoantibodies, rheumatoid factor, and serum gammaglobulin and IgG levels. Likewise, the absence of genetic involvement of BAFF in RA or SLE was reported in Japanese patients [7]. To a greater extent, no association was observed between polymorphisms in BCMA [14], TACI [15] and BAFF receptors, and RA or SLE. This suggests that autoimmunity does not result from a primary genetically determined activation of the BAFF/BAFF receptor system, in contrast to the recent demonstration of the genetic association between common variable immunodeficiency and TACI [16]. Like in mouse models of autoimmunity, BAFF over-expression might amplify an independent genetically determined autoimmune proclivity, rather than creating an autoimmune disease *de novo *[17].

Interestingly, among the 26 individuals (13 patients with pSS and 13 control individuals) who were entirely sequenced for the BAFF gene, the only SNP detected at a significant frequency in the present study was located in the promoter region of BAFF. Moreover, this promoter polymorphism lies in a putative binding site for nuclear factor-κB, which is known to enhance BAFF gene expression. To date, the only data available regarding the functional role of BAFF polymorphism are derived from a Japanese study that included 12 healthy individuals [7] and reported a significant association between -871 T allele and increased BAFF mRNA level in blood monocytes. We therefore investigated the association between BAFF -871 T/C polymorphism and BAFF expression, and focused first on the correlation between BAFF polymorphism and serum level as assessed by enzyme-linked immunosorbent assay. Interestingly, serum BAFF protein level was high in patients carrying two -871 T alleles, intermediate in patients with one T allele, and low in patients without a T allele. It is remarkable to find such an association in a cross-sectional study because BAFF level could have been modulated by disease activity and perhaps by treatment. BAFF serum level remained correlated with T allele even in patients without anti-SSA/SSB autoantibodies, in whom disease is usually less systemic. Moreover, we previously reported that BAFF level was not associated with systemic features in pSS [8]. Likewise, no association was found between BAFF level and disease activity in SLE patients [12]. Moreover, no significant change in serum BAFF level was observed in our patients treated with low-dose corticosteroids or hydroxychloroquine. Accordingly, in a longitudinal study [12] it was found that the BAFF protein level was stable in 74% of patients with SLE and that immunosuppressive medications (except high-dose corticosteroids, which was never prescribed to our patients) did not influence BAFF level.

Surprisingly, no correlation was observed between BAFF polymorphism and BAFF mRNA levels in patients with pSS. The absence of concordance between BAFF protein and mRNA regarding the correlation with BAFF polymorphism might be related to the fact that assessments of BAFF mRNA and protein were not performed on the same day. Despite this limitation, our findings suggest that BAFF mRNA does not correlate with protein level in some patients with pSS. Interestingly, a longitudinal study evaluating BAFF levels also reported that the BAFF mRNA phenotype did not match the BAFF protein phenotype in as many as 42% of patients with SLE, with reciprocal changes between mRNA and protein levels in 21% of patients [12]. In autoimmune diseases there might be some feedback regulatory mechanism through which the increase in circulating levels of BAFF protein leads to downregulation of BAFF transcription. This might contribute to a transient dissociation between BAFF protein and BAFF mRNA levels. Saturation of BAFF receptors and/or a downregulation of their expression in patients with increased BAFF levels might further amplify the increase in BAFF protein levels. More speculatively, a decrease in ΔBAFF protein, which inhibits secretion of BAFF [18], would also increase BAFF protein level without affecting BAFF mRNA level. The absence of concordance between BAFF protein and mRNA regarding the correlation with BAFF polymorphism precludes any definitive conclusion regarding the functional role of this polymorphism. To provide direct evidence that -871 T polymorphism of the BAFF promoter gene is associated with production of BAFF protein, analysis of BAFF expression must be performed using transfectant expressing -871 T in the promoter gene. Likewise, the functional difference between -871 T and -871 C on transcription factor binding should be investigated using luciferase assay or electrophoretic mobility shift assay.

## Conclusion

The association of BAFF polymorphism with BAFF levels requires further investigation. The increase in BAFF level in pSS might be under the control of environmental factors. Interestingly, BAFF gene expression was reported to be interferon inducible in target organs of patients with RA [19] and multiple sclerosis [20]. Moreover, pathogenic activation of interferon signalling pathways was recently demonstrated in salivary glands of patients with pSS [21,22]. Thus, the role played by interferons in BAFF over-expression in pSS deserves further investigation. Finally, our study clearly demonstrates that BAFF gene polymorphism is neither involved in genetic predisposition to pSS nor associated with a specific pattern of antibody production.

## Abbreviations

BAFF = B-cell-activating factor; CI = confidence interval; PBMC = peripheral blood mononuclear cell; pSS = primary Sjögren's syndrome; RA = rheumatoid arthritis; RT-PCR = reverse transcriptase polymerase chain reaction; SLE = systemic lupus erythematosus; SNP = single nucleotide polymorphism.

## Competing interests

The authors declare that they have no competing interests.

## Authors' contributions

JEG and CMR carried out molecular genetic studies. XM and CM-R designed the study, contributed to acquisition of clinical data, and analyzed and interpreted the data. JS, MI, FL, AP, HZ, CS, JS and RP performed acquisition of data.

## Figures and Tables

**Table 1:** Characteristics of the population studied

Characteristic	Value
Age (mean ± SD)	57.3 ± 12.1
Women/men (*n*)	156/6
No anti-SSA or anti-SSB autoantibodies (*n *[%])	52 (32)
Anti-SSA autoantibody only (*n *[%])	54 (33.4)
Anti-SSA and anti-SSB autoantibodies (*n *[%])	56 (34.6)
Positive rheumatoid factor finding (*n *[%])	89 (55)
Rheumatoid factor level (IU/l; mean ± SD)	183.6 ± 365
Serum IgG level (g/l; mean ± SD)	15 ± 7.2
Focus score ≥1 on labial salivary gland (*n *= 145; *n *[%])	130 (90)

SD, standard deviation.
